# Alcohol consumption and the risk of heart failure: the Suita Study and meta-analysis of prospective cohort studies

**DOI:** 10.1265/ehpm.22-00231

**Published:** 2023-05-03

**Authors:** Ahmed Arafa, Rena Kashima, Yoshihiro Kokubo, Masayuki Teramoto, Yukie Sakai, Saya Nosaka, Haruna Kawachi, Keiko Shimamoto, Chisa Matsumoto, Qi Gao, Chisato Izumi

**Affiliations:** 1 Department of Preventive Cardiology, National Cerebral and Cardiovascular Center, Suita, Japan; 2 Department of Public Health, Faculty of Medicine, Beni-Suef University, Beni-Suef, Egypt; 3 Department of Cardiovascular Pathophysiology and Therapeutics, Graduate School of Medicine, Osaka University, Suita, Japan; 4 Department of Environmental Medicine and Population Sciences, Graduate School of Medicine, Osaka University, Suita, Japan; 5 Department of Cardiovascular Medicine, National Cerebral and Cardiovascular Center, Suita, Japan; 6 Department of Cardiology, Center for Health Surveillance and Preventive Medicine, Tokyo Medical University Hospital, Shinjuku, Japan; 7 Department of Heart Failure and Transplantation, National Cerebral and Cardiovascular Center, Suita, Japan

**Keywords:** Alcohol consumption, Heart failure, Prospective cohort, Japan, Meta-analysis

## Abstract

**Background:**

Alcohol consumption is a modifiable lifestyle, but its role in heart failure (HF) development is controversial. Herein, we investigated the prospective association between alcohol consumption and HF risk.

**Methods:**

A total of 2,712 participants (1,149 men and 1,563 women) from the Suita Study were followed up every two years. Cox regression was applied to calculate the hazard ratios (HRs) and 95% confidence intervals (95% CIs) of HF risk for heavy drinking (≥46 g/day in men or ≥23 g/day in women) and never drinking compared to light drinking (<23 g/day in men or <11.5 g/day in women). Then, we combined the results of the Suita Study with those from other eligible prospective cohort studies in a meta-analysis using the random-effects model.

**Results:**

In the Suita Study, within a median follow-up period of 8 years, 319 HF cases (162 in men and 157 in women) were detected. In men, but not women, never and heavy drinking carried a higher risk of HF than light drinking: HRs (95% CIs) = 1.65 (1.00, 2.73) and 2.14 (1.26, 3.66), respectively. Alike, the meta-analysis showed a higher risk of HF among heavy drinkers: HR (95% CI) = 1.37 (1.15, 1.62) and abstainers: HR (95% CI) = 1.18 (1.02, 1.37).

**Conclusion:**

We indicated a J-shaped association between alcohol consumption and HF risk among Japanese men. The results of the meta-analysis came in line with the Suita Study. Heavy-drinking men should be targeted for lifestyle modification interventions.

**Supplementary information:**

The online version contains supplementary material available at https://doi.org/10.1265/ehpm.22-00231.

## 1. Introduction

Heart failure (HF) is a major public health concern in Japan and worldwide. In addition to its grave consequences, HF is estimated to seize 1–2% of total healthcare budgets [1–3]. Since HF is potentially preventable, identifying at-risk individuals with the aim of lifestyle modification interventions is promoted [4, 5].

Among modifiable lifestyle factors, alcohol consumption appears to play a role in the development of cardiovascular disease (CVD), including HF [6–9]. Heavy alcohol consumption could induce myocardial damage and impaired left ventricular function, factors that eventually result in alcoholic cardiomyopathy and HF [10–12].

Several studies have examined the association between alcohol consumption and HF risk [13–31]. Apart from their inconsistent findings, many of these studies were limited by 1) the use of alcohol abstinence as a reference group [13–23] that could hide the hazardous impact of heavy drinking on HF [32], 2) involving former drinkers who posed unfavorable clinical profile in the alcohol abstinence group [14, 16, 22, 25], 3) applying low cut-offs of alcohol consumption to define heavy drinking which was insufficient to detect a significant association and carried high risks of misclassification [14–16, 19–22, 25], and 4) underrepresenting Asian populations although the contribution of alcohol consumption to CVD risk may vary across races [33].

We, therefore, used data from the Suita Study, a prospective cohort study conducted in urban Japan, to examine the association between alcohol consumption and HF risk. We avoided the main limitation of previous studies by assigning light drinking as a reference group to avoid false negative results. We also stratified the results by several personal and clinical factors to help personalize future preventive measures. Then, we performed a meta-analysis combining the results of the Suita Study with those from other eligible cohort studies to confirm our findings.

## 2. Methods

### 2.1. The Suita Study

#### 2.1.1. Study population and design

The Suita Study is a population-based study with a prospective cohort design. Middle-aged and older adults residing in the urban city of Suita in Southwest Japan were randomly recruited by age category and sex. A baseline assessment, in the form of an interview, clinical examination, blood sample collection, and ECG, was performed at the National Cerebral and Cardiovascular Center (NCVC) in Suita. Then, participants were asked to attend follow-ups every couple of years at the same place [34–39].

In this study, a total of 3,573 participants (1,590 men and 1,983 women), aged 40–92 years, were included. However, we excluded participants with critical missing data (n = 5), participants who quit drinking (n = 140), participants with a previous history of HF or brain natriuretic peptide (BNP) levels ≥ 100 pg/mL (n = 181), or participants who were lost to follow-up (n = 535), leaving 2,712 participants (1,149 men and 1,563 women) for analysis. In both sexes, those who were lost to follow-up were significantly older and included higher proportions of CVD risk factors than participants in the analysis group (Supplementary file 1).

#### 2.1.2. Assessment of alcohol consumption

Alcohol consumption was assessed using a baseline questionnaire administered by an interviewer. Participants were asked about the frequency of alcohol consumption during a typical week and the amount of alcohol consumed on each occasion. Alcohol consumption was measured by *gou*, a traditional Japanese measurement unit expressing an amount of alcohol equivalent to two drinks or 23 g of pure alcohol [39]. In the current study, light, moderate, and heavy alcohol consumption was defined as daily drinking equivalent to <one *gou* in men or <0.5 *gou* in women, ≥one but <two *gou* in men or ≥0.5 but <one *gou* in women, and ≥two *gou* in men or ≥one *gou* in women, respectively.

#### 2.1.3. Diagnosis of heart failure

As described elsewhere [38], HF was identified if participants developed BNP levels ≥ 100 pg/mL during the follow-ups that were conducted at the NCVC every two years or were diagnosed with HF by their physicians as shown in their medical records. The Japanese Heart Failure Society (JHFS) suggested the applied BNP cut-off as a likely indicator for HF [40]. The diagnostic accuracy of BNP in HF was confirmed in a previous meta-analysis [41]. BNP, in this study, was measured using the Chemiluminescent Enzyme Immunoassay (CLEIA) as described elsewhere [42].

#### 2.1.4. Statistical analyses

We used the SAS version 9.4 software (SAS Institute Inc, Cary, NC) for statistical analyses. The differences in baseline characteristics of participants by their alcohol consumption (never, light, moderate, and heavy) were assessed by the Chi-squared test for categorical variables and one-way ANOVA for continuous variables. Then, we performed the Cox proportional hazards models to calculate the age-adjusted and the multivariable-adjusted hazard ratios (HRs) and their 95% confidence intervals (95% CIs) of HF risk for never, moderate, and heavy drinkers compared to light drinkers in the overall population and population with no preceding stroke or coronary heart disease (CHD). Person-years of follow-up were calculated from baseline examination (between 2005 and 2016) to the date of HF diagnosis, death, censoring, or the last health examination (up to 2020), whichever came first.

We further stratified the results by age group (<70 and ≥70 years), body mass index (BMI) (<25 and ≥25 kg/m^2^), smoking behavior (never and ever), blood pressure (<140/90 and ≥140/90 mmHg), hypertension medication (yes and no), and high-density lipoprotein (HDL) (<50 and ≥50 mg/dL in men and <60 and ≥60 mg/dL in women), and the corresponding p-interactions were computed. All analyses were stratified by sex.

The following baseline characteristics were included in the Cox regression models: age (continuous), BMI (continuous), smoking (never, former, or current), systolic and diastolic blood pressure (continuous), hypertension medication (yes or no), fasting blood glucose (FBG) (continuous), glomerular filtration rate (GFR) (continuous), HDL (continuous), lipid-lowering agent use (yes or no), arrhythmia including atrial fibrillation (yes or no), cardiac murmurs or valvular diseases (yes or no), and preceding stroke or CHD (yes or no).

### 2.2. The meta-analysis

#### 2.2.1. Literature search

The first-and second-place authors searched MEDLINE (PubMed) and Scopus for potential studies published in English before the 15^th^ of June 2022 (the last day of the data search) using the following terms: (Alcohol) AND (Heart failure). The search strategy was provided (Supplementary file 2). In addition, we manually searched the reference lists of the selected articles to obtain additional relevant studies. We reported this meta-analysis per the checklists of the Preferred Reporting Items for Systematic Reviews and Meta-Analysis (PRISMA) [43] and Meta-analyses Of Observational Studies in Epidemiology (MOOSE) [44].

#### 2.2.2. Study selection

Studies abiding by the following criteria were selected for the meta-analysis: 1) heavy alcohol drinking and alcohol abstinence were the exposures, 2) light or moderate drinking was the reference group, 3) HF was the outcome, and 4) the study had a prospective cohort design. No limits were set regarding the year of publication, but we did not manage to obtain unpublished data. The following relevant information was extracted from the selected studies: study ID (last name of the first author, study name and place, and publication year), frequency, age, and sex of the study populations, alcohol consumption cut-offs for heavy drinking and the reference group, follow-up years, number of HF incident cases, and covariates included in the most adjusted regression models. We assessed the quality of studies per the modified Newcastle-Ottawa Quality Assessment Scale (NOS) based on studies’ selection (representativeness, selection of the non-exposed, ascertainment of the exposure, and demonstration of the outcome), comparability, and outcome (assessment, follow-up length, and adequacy) [45].

#### 2.2.3. Statistical analysis

We applied the random-effects model to compute the pooled HR with 95% CI of HF risk for heavy drinking and alcohol abstinence compared to light or moderate drinking [46], the *I*^2^ statistic to measure heterogeneity across studies [47], and the regression test for funnel plot asymmetry to assess publication bias [48]. To test the impact of each study, we removed studies one by one and combined the remaining studies in separate meta-analyses. We used the R-3.2.0 statistical package (Metafor: Meta-Analysis Package for R) for meta-analysis [49].

## 3. Results

### 3.1. The Suita Study

Among the 1,149 included men, 340 (29.6%) were never drinkers, 212 (18.5%) were light drinkers, 291 (25.3%) were moderate drinkers, and 306 (26.6%) were heavy drinkers. Among the 1,563 included women, 1,162 (74.4%) were never drinkers, 94 (6.0%) were light drinkers, 155 (9.9%) were moderate drinkers, and 152 (9.7%) were heavy drinkers. Men and women who reported heavy drinking were more likely to be current smokers, but they were significantly younger and had higher HDL levels (p < 0.05) (Table 1).

**Table 1:** Characteristics of participating men and women by alcohol consumption

**Characteristics**		**Alcohol consumption**				**P-value**

		**Never**	**Light**	**Moderate**	**Heavy**	
Men						
Frequency		340	212	291	306	—
Age, years*		68.4 ± 10.5	69.3 ± 10.8	68.0 ± 9.7	63.3 ± 8.8	<0.001
Body mass index, kg/m^2 ^*		23.3 ± 3.1	23.5 ± 2.7	23.3 ± 2.5	23.5 ± 3.0	0.055
Smoking, %	Never	35.9	28.8	21.3	17.3	<0.001
	Former	44.1	54.7	61.5	44.5	
	Current	20.0	16.5	17.2	38.2	
Systolic blood pressure, mmHg*		128.6 ± 18.2	129.1 ± 17.9	130.2 ± 18.0	131.9 ± 18.3	0.975
Diastolic blood pressure, mmHg*		78.8 ± 10.5	79.4 ± 10.1	79.9 ± 9.7	83.1 ± 10.4	0.525
Hypertension medication, %		30.6	26.9	36.8	32.7	0.113
Fasting blood glucose, mg/dL*		107.6 ± 26.2	106.0 ± 23.2	108.6 ± 23.4	109.5 ± 22.8	0.871
Glomerular filtration rate, ml/min/1.73 m^2 ^*		72.7 ± 16.8	73.0 ± 15.1	72.8 ± 14.7	78.3 ± 14.8	0.232
High-density lipoprotein-cholesterol, mg/dL*		50.8 ± 12.5	54.2 ± 13.5	57.3 ± 14.3	61.5 ± 16.4	0.006
Lipid-lowering agent, %		17.4	12.7	16.5	10.1	0.037
Cardiac murmur or valvular disease, %		5.9	8.5	7.6	4.6	0.259
Arrhythmia including atrial fibrillation, %		16.8	18.9	14.4	12.8	0.233
Preceding stroke or coronary heart disease, %		9.4	7.6	8.9	7.5	0.786
Women						
Frequency		1,162	94	155	152	—
Age, years*		66.7 ± 10.0	66.1 ± 9.9	61.3 ± 8.2	60.4 ± 9.1	<0.001
Body mass index, kg/m^2 ^*		22.3 ± 3.4	21.8 ± 2.6	22.0 ± 2.8	22.4 ± 3.1	0.199
Smoking, %	Never	92.4	93.6	82.6	71.1	<0.001
	Former	4.6	4.3	11.6	10.5	
	Current	3.0	2.1	5.8	18.4	
Systolic blood pressure, mmHg*		125.9 ± 20.6	124.5 ± 17.9	121.0 ± 19.8	123.9 ± 19.2	0.032
Diastolic blood pressure, mmHg*		74.8 ± 11.0	75.9 ± 10.4	73.6 ± 11.6	76.2 ± 11.6	0.644
Hypertension medication, %		26.8	23.4	18.1	27.6	0.114
Fasting blood glucose, mg/dL*		99.4 ± 14.8	97.6 ± 9.0	96.8 ± 10.2	98.6 ± 12.3	0.031
Glomerular filtration rate, ml/min/1.73 m^2 ^*		76.4 ± 16.1	76.5 ± 15.1	79.9 ± 13.0	80.6 ± 14.8	0.001
High-density lipoprotein-cholesterol, mg/dL*		64.4 ± 14.5	66.2 ± 12.9	69.5 ± 15.6	72.5 ± 15.8	<0.001
Lipid-lowering agent, %		24.4	21.3	12.3	11.8	<0.001
Cardiac murmur or valvular disease, %		8.4	8.5	3.9	4.6	0.108
Arrhythmia including atrial fibrillation, %		13.3	16.0	9.7	5.9	0.032
Preceding stroke or coronary heart disease, %		5.3	2.1	0.0	1.3	0.003

*Mean ± standard deviation for all such variables

In men, within 8,619 person-years (median follow-up = 8 years), 162 HF cases were detected. The incidence of HF / 1,000 person-years was distributed as follows: 20.5 in never drinkers, 16.1 in light drinkers, 19.0 in moderate drinkers, and 18.6 in heavy drinkers. Compared to light drinkers, heavy and never drinkers showed a higher risk of HF in the multivariable-adjusted models: HRs (95% CIs) = 2.14 (1.26, 3.66) and 1.65 (1.00, 2.73), respectively. Moderate drinkers showed a slightly higher HF risk than light drinkers, but this rise was statistically insignificant. However, in current drinkers, the amount of alcohol consumed was not linearly associated with HF risk. Among participants with no preceding stroke or CHD, the risk of HF in heavy and never drinkers, like the overall population, was elevated: HRs (95% CIs) = 2.41 (1.33, 4.37) and 1.74 (1.01, 3.00), respectively. Yet, unlike the overall population, every 23 g/day increase in alcohol consumption was associated with a 19% increase in HF risk. In women, within 12,059 person-years (median follow-up = 8 years), 157 HF cases were detected. The incidence of HF / 1,000 person-years was distributed as follows: 13.8 in never drinkers, 13.6 in light drinkers, 12.1 in moderate drinkers, and 8.6 in heavy drinkers. Unlike men, women who reported heavy and never drinking showed no excess HF risk (Table 2).

**Table 2:** Association between alcohol consumption and heart failure risk in Japanese men and women

	**Alcohol consumption**				**P for trend**	Per 23 g/day increase*

	**Never**	**Light**	**Moderate**	**Heavy**		
**All men**						
Incident cases	50	25	42	45	—	—
Incidence / 1,000 person-years	20.5	16.1	19.0	18.6	—	—
Model I, HR (95% CI)	1.48 (0.91, 2.41)	1 (Ref)	1.38 (0.84, 2.29)	2.40 (1.44, 4.02)	0.008	1.03 (0.97, 1.09)
Model II, HR (95% CI)	1.62 (0.99, 2.67)	1 (Ref)	1.31 (0.79, 2.19)	2.24 (1.31, 3.83)	0.026	1.02 (0.96, 1.09)
Model III, HR (95% CI)	1.65 (1.00, 2.73)	1 (Ref)	1.27 (0.76, 2.12)	2.14 (1.26, 3.66)	0.019	1.01 (0.94, 1.08)
**Men with no preceding stroke or coronary heart disease**						
Incident cases	43	21	32	39	—	—
Incidence / 1,000 person-years	19.1	14.5	15.7	17.1	—	—
Model I, HR (95% CI)	1.55 (0.91, 2.65)	1 (Ref)	1.31 (0.75, 2.30)	2.60 (1.48, 4.57)	0.011	1.24 (1.06, 1.45)
Model II, HR (95% CI)	1.72 (1.00, 2.97)	1 (Ref)	1.20 (0.68, 2.13)	2.22 (1.23, 4.00)	0.032	1.19 (0.99, 1.43)
Model III, HR (95% CI)	1.74 (1.01, 3.00)	1 (Ref)	1.27 (0.72, 2.26)	2.41 (1.33, 4.37)	0.018	1.19 (1.00, 1.42)
**All women**						
Incident cases	120	10	16	11	—	—
Incidence / 1,000 person-years	13.8	13.6	12.1	8.6	—	—
Model I, HR (95% CI)	0.78 (0.41, 1.50)	1 (Ref)	1.22 (0.55, 2.70)	1.01 (0.43, 2.40)	0.931	1.06 (0.94, 1.19)
Model II, HR (95% CI)	0.79 (0.42, 1.52)	1 (Ref)	1.24 (0.56, 2.76)	0.98 (0.41, 2.35)	0.952	1.06 (0.93, 1.20)
Model III, HR (95% CI)	0.73 (0.38, 1.41)	1 (Ref)	1.24 (0.56, 2.76)	0.95 (0.40, 2.30)	0.987	1.06 (0.94, 1.19)
**Women with no preceding stroke or coronary heart disease**						
Incident cases	106	9	16	11	—	—
Incidence / 1,000 person-years	12.7	12.3	12.1	8.7	—	—
Model I, HR (95% CI)	0.81 (0.41, 1.60)	1 (Ref)	1.34 (0.59, 3.04)	1.17 (0.48, 2.83)	0.715	1.07 (0.96, 1.18)
Model II, HR (95% CI)	0.83 (0.42, 1.65)	1 (Ref)	1.34 (0.59, 3.08)	1.11 (0.45, 2.75)	0.750	1.06 (0.94, 1.20)
Model III, HR (95% CI)	0.81 (0.41, 1.61)	1 (Ref)	1.37 (0.59, 3.14)	1.14 (0.46, 2.83)	0.724	1.06 (0.95, 1.19)

*In current drinkers only (23 g of alcohol is equivalent to 1 gou or approximately 2 drinks)

Model I: Adjusted for age

Model II: Adjusted further for body mass index, smoking, systolic and diastolic blood pressure, hypertension medication, fasting blood glucose, glomerular filtration rate, high-density lipoprotein-cholesterol, and lipid-lowering agent

Model III: Adjusted further for cardiac murmur or valvular disease, arrhythmia, and preceding stroke or coronary heart disease

P-interaction for preceding stroke or coronary heart disease = 0.398 in men and 0.985 in women

In the stratified analysis, the risk of HF was more prominent among men with BMI ≥ 25 kg/m^2^ than their counterparts with BMI < 25 kg/m^2^; HRs (95% CIs) = 2.27 (0.68, 7.54) versus 1.69 (0.96, 2.99) in never drinkers, 3.01 (0.95, 9.53) versus 1.05 (0.58, 1.90) in moderate drinkers, and 3.73 (1.14, 12.22) versus 2.03 (1.08, 3.84) in heavy drinkers, respectively (p-interaction = 0.014). Besides, the association between heavy drinking and HF risk was more robust among ever-smoking than never-smoking men; HRs (95% CIs) = 2.33 (1.27, 4.27) and 1.69 (0.54, 5.26), respectively (p-interaction = 0.106) (Table 3).

**Table 3:** Multivariable-adjusted associations between alcohol consumption and heart failure risk by several factors

**Variables**		**Alcohol consumption**				**P-interaction**

		**Never**	**Light**	**Moderate**	**Heavy**	
Men						
Age (years)	<70	1.25 (0.42, 3.69)	1 (Ref)	1.66 (0.58, 4.80)	2.00 (0.71, 5.60)	0.698
	≥70	1.87 (1.04, 3.39)	1 (Ref)	1.14 (0.62, 2.08)	2.17 (1.12, 4.20)	
Body mass index (kg/m^2^)	<25	1.69 (0.96, 2.99)	1 (Ref)	1.05 (0.58, 1.90)	2.03 (1.08, 3.84)	0.014
	≥25	2.27 (0.68, 7.54)	1 (Ref)	3.01 (0.95, 9.53)	3.73 (1.14, 12.22)	
Smoking	Never	2.16 (0.85, 5.49)	1 (Ref)	1.14 (0.37, 3.54)	1.69 (0.54, 5.26)	0.106
	Ever	1.50 (0.81, 2.77)	1 (Ref)	1.33 (0.74, 2.37)	2.33 (1.27, 4.27)	
Blood pressure (mmHg)	<140/90	1.67 (0.85, 3.31)	1 (Ref)	1.70 (0.85, 3.43)	3.02 (1.47, 6.22)	0.678
	≥140/90	1.62 (0.74, 3.54)	1 (Ref)	0.97 (0.46, 2.06)	1.56 (0.69, 3.53)	
Hypertension medication	Yes	2.05 (0.88, 4.77)	1 (Ref)	1.38 (0.59, 3.25)	1.74 (0.70, 4.34)	0.196
	No	1.27 (0.67, 2.41)	1 (Ref)	1.10 (0.58, 2.10)	2.51 (1.27, 4.97)	
High-density lipoprotein (mg/dL)	≥50	1.61 (0.82, 3.17)	1 (Ref)	1.66 (0.86, 3.19)	2.40 (1.24, 4.64)	0.706
	<50	1.55 (0.73, 3.29)	1 (Ref)	0.91 (0.37, 2.23)	2.07 (0.77, 5.60)	
Women						
Age (years)	<70	1.74 (0.41, 7.35)	1 (Ref)	2.54 (0.54, 12.05)	1.95 (0.37, 10.14)	0.604
	≥70	0.52 (0.25, 1.10)	1 (Ref)	1.07 (0.38, 3.01)	0.88 (0.28, 2.83)	
Body mass index (kg/m^2^)	<25	0.77 (0.37, 1.60)	1 (Ref)	1.63 (0.68, 3.90)	0.78 (0.28, 2.21)	0.588
	≥25	0.20 (0.04, 1.01)	1 (Ref)	0.09 (0.01, 1.15)	0.59 (0.08, 4.15)	
Smoking	Never	0.76 (0.38, 1.51)	1 (Ref)	1.10 (0.45, 2.67)	0.92 (0.34, 2.50)	0.018
	Ever	0.14 (0.01, 3.78)	1 (Ref)	1.05 (0.04, 25.25)	0.53 (0.02, 13.94)	
Blood pressure (mmHg)	<140/90	1.38 (0.50, 3.79)	1 (Ref)	2.45 (0.77, 7.79)	2.10 (0.61, 7.22)	0.631
	≥140/90	0.28 (0.11, 0.72)	1 (Ref)	0.47 (0.13, 1.70)	0.29 (0.07, 1.23)	
Hypertension medication	Yes	0.36 (0.14, 0.89)	1 (Ref)	0.47 (0.12, 1.80)	1.25 (0.39, 4.02)	0.062
	No	1.18 (0.42, 3.30)	1 (Ref)	2.07 (0.66, 6.53)	0.65 (0.14, 3.05)	
High-density lipoprotein (mg/dL)	≥60	0.66 (0.31, 1.40)	1 (Ref)	1.37 (0.56, 3.39)	0.91 (0.34, 2.43)	0.309
	<60	0.82 (0.19, 3.56)	1 (Ref)	0.87 (0.14, 5.58)	1.09 (0.13, 9.26)	

### 3.2. The meta-analysis

**Fig. 1:**
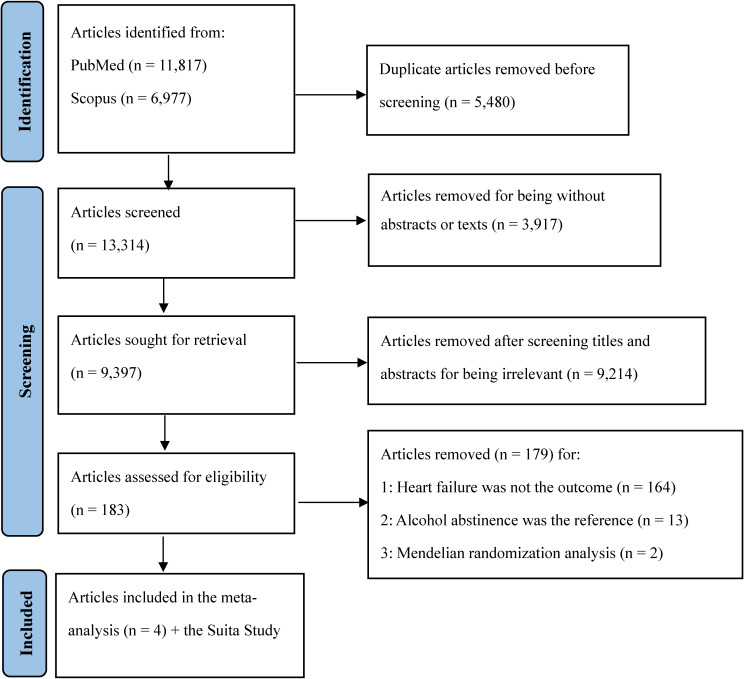
PRISMA flow diagram of the study selection process for the meta-analysis

After removing duplicates, review articles, and irrelevant studies, a shortlist of 183 studies was made. We further removed 179 studies for not investigating HF (n = 164), assigning alcohol abstinence as a reference group (n = 13), and performing a Mendelian randomization analysis (n = 2). Eventually, four studies (five cohorts) were found eligible [24, 27–29], and their results were combined with those from the Suita Study (two cohorts) in this meta-analysis (Fig. 1). The included studies used data from the British Regional Heart Study (BRHS) [24], the British ClinicAl research using LInked Bespoke studies and Electronic health Records (CALIBER) [27], the Cohort of Swedish Men (COSM) [28], the Swedish Mammography Cohort (SMC) [28], 83 cohorts from 19 high-income countries [29], and the Japanese Suita Study (Table 4). All studies were of good quality per the modified NOS (Supplementary file 3).

**Table 4:** Summary of the studies included in the meta-analysis

**Study ID**	**Population**	**Drinking**	**Follow-up**	**Incident HF**	**Covariates**
Wannamethee et al. (2015) BRHS, UK	n = 3,530 60–79 years Men	Heavy: ≥35 drinks/week Ref: 1–6 drinks/week	11 years Mean	n = 120	1, 3, 4, 5, 6, 7, 8, 9, 10
Bell et al. (2017) CALIBER, UK	n = 1,937,360 ≥30 years Men and women No cardiovascular disease	Heavy: ≥21 drinks/week in men and ≥14 drinks/week in women Ref: <21 drinks/week in men and <14 drinks/week in women	6 years Median	n = 14,359	1, 2, 3, 4, 5, 6, 7
Larsson et al. (2017) COSM, Sweden	n = 40,590 45–79 years Men	Heavy: >28 drinks/week Ref: <1 drink/week	11.9 years Mean	n = 1,905	1, 3, 4, 5, 6, 7, 8, 9, 10
Larsson et al. (2017) SMC, Sweden	n = 34,022 49–83 years Women	Heavy: >21 drinks/week Ref: <1 drink/week	12.2 years Mean	n = 1,328	1, 3, 4, 5, 6, 7, 8, 9, 10
Wood et al. (2018) 83 cohorts, 19 countries	n = 670,923 Mean age: 50s Men and women No cardiovascular disease	Heavy: 300 g/week Ref: <25 g/week	7.5 years Mean	n = 2,711	1, 2, 4, 6
Arafa et al. (2022) Suita Study, Japan	n = 2,712 40–92 years Men and women	Heavy: ≥2 gou/day in men and ≥1 gou/day in women Ref: <1 gou/day in men and <0.5 gou/day in women	8 years Median	n = 319	1, 3, 4, 5, 6, 7, 8, 9, 10

Covariates: 1- age, 2- sex, 3- body mass index, 4- smoking, 5- hypertension, 6- diabetes, 7- dyslipidemia, 8- chronic kidney disease, 9- arrhythmia, 10- preceding or incident stroke or coronary heart disease

**Fig. 2:**
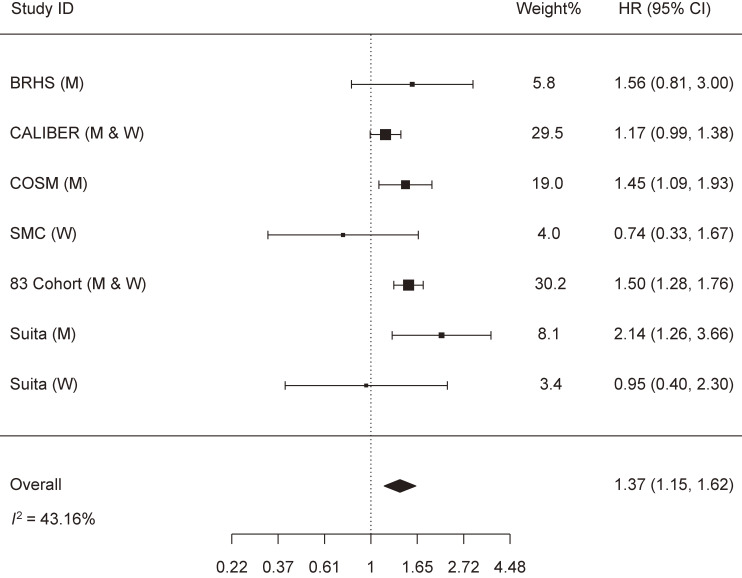
Meta-analysis of the association between heavy drinking and heart failure risk

The meta-analysis that assessed the relationship between heavy drinking and HF risk included seven cohorts with the following weights: BRHS (5.8%), CALIBER (29.5%), COSM (19.0%), SMC (4.0%), 83 Cohort (30.2%), and the Suita Study in men (8.1%) and in women (3.4%). Heavy drinking was associated with the increased risk of HF: HR (95% CI) = 1.37 (1.15, 1.62). A moderate heterogeneity across studies was detected (*I*^2^ = 43.16% and p-heterogeneity = 0.103) (Fig. 2). Removing the CALIBER study significantly minimized the heterogeneity (*I*^2^ = 18.72%) and modestly strengthened the association: HR (95% CI) = 1.44 (1.21, 1.72) (Supplementary file 4). We detected no publication bias (Z = −0.392, p-publication bias = 0.695) (Supplementary file 5).

**Fig. 3:**
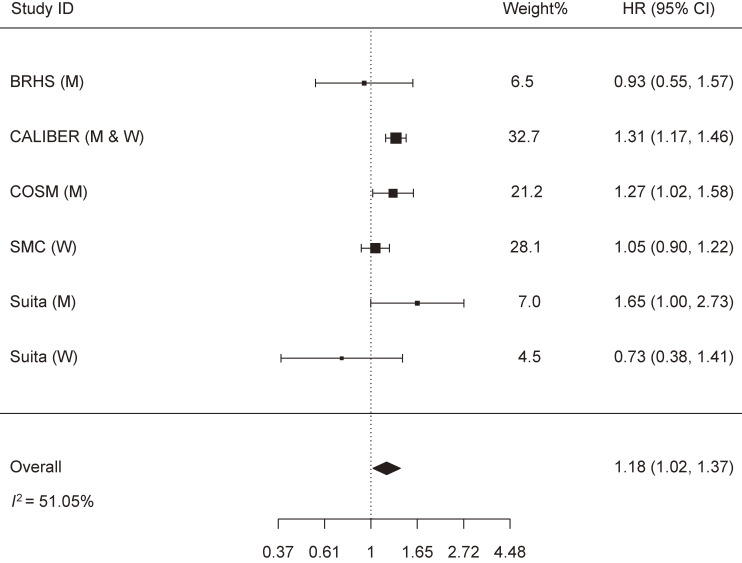
Meta-analysis of the association between alcohol abstinence and heart failure risk

The meta-analysis that assessed the relationship between alcohol abstinence and HF risk included six cohorts with the following weights: BRHS (6.5%), CALIBER (32.7%), COSM (21.2%), SMC (28.1%), and the Suita Study in men (7.0%) and in women (4.5%). It should be noted that the studies of CALIBER, COSM, SMC, and Suita confined their alcohol abstinence group to never-drinkers, while the BRHS study included non-drinkers who might have included former drinkers. Alcohol abstinence was significantly associated with increased HF risk: HR (95% CI) = 1.18 (1.02, 1.37). The heterogeneity across studies was moderate (*I*^2^ = 51.05% and p-heterogeneity = 0.069) (Fig. 3). Removing the SMC study almost halved the heterogeneity (*I*^2^ = 25.88%), while the associations remained significant: HR (95% CI) = 1.26 (1.08, 1.45) (Supplementary file 4). No publication bias was identified (Z = −0.743, p-publication bias = 0.457) (Supplementary file 6).

## 4. Discussion

The Suita Study indicated higher HF risk in heavy and never drinking men than light drinkers by 114% and 65%, respectively. A similar pattern was detected in the meta-analysis which included the prospective cohort studies that assigned light or moderate drinkers as a reference group; the HF risk increased by 37% in heavy drinkers and 18% in abstainers. Our findings reflect the need to consider alcohol consumption in future HF risk scores and target heavy drinkers for screening and intervention.

Previous studies assessing the relationship between alcohol consumption and HF risk investigated Western populations and reached inconsistent findings. The Established Populations for the Epidemiologic Study on the Elderly program (EPESE) [14], the Framingham Heart Study [15], the Cardiovascular Health Study (CHS) [18], the Physicians’ Health Study I (PHS I) [19, 20], the Stockholm Heart Epidemiology Program (SHEEP) [21], the Atherosclerosis Risk in Communities Study (ARIC) [23], and the Nord-Trøndelag Health Study (HUNT) [25] indicated that drinking in moderation could carry protective effects against HF risk, while heavy drinking was not associated with the increased risk of HF. However, these studies assigned abstainers as a reference group [14, 15, 18–21, 23, 25], and many of them used low cut-offs to define the highest category of alcohol consumption [14, 19–21, 25]. A meta-analysis of eight prospective cohort studies reported that compared to abstainers, light-to-moderate drinking led to a 15% reduction in HF risk, but heavy drinking did not elevate HF risk [50]. On the other hand, heavy drinking was positively associated with HF risk in the BRHS [24], CALIBER [27], and COSM [28] studies. The increased HF risk with alcohol abstinence was reported in the studies of CALIBER [27] and COSM [28] but not in BRHS [24]. The three studies assigned light or moderate drinkers as a reference group and used high cut-offs of alcohol consumption to define heavy drinking. Importantly, when we repeated the analysis in the Suita Study with never-drinkers assigned as a reference group, the association between heavy drinking and HF risk disappeared (data not shown). When we conducted a meta-analysis on studies that assigned alcohol abstinence as a reference group, no association was detected between heavy drinking and HF risk (data not shown). Therefore, we believe that the main reason for not detecting the impact of heavy drinking on HF risk in previous studies was assigning abstainers, who are at higher risk of HF than light drinkers, as a reference group.

In line with our findings, several Japanese epidemiological studies revealed J- or U-shaped associations between alcohol consumption and CVD among men. A study including 34,776 men (40–79 years) from the Japan Collaborative Cohort Study (JACC) showed increased CVD mortality among heavy drinkers and decreased CVD mortality among light-to-moderate drinkers [51]. Another study including 2,890 men (40–69 years) from three rural communities in Japan showed a J-shaped relationship between alcohol consumption and the risk of non-hemorrhagic stroke [52]. In 2,336 men (30–79 years) from the Suita Study, a U-shaped association between alcohol consumption and the risk of CVD and CHD was detected in hypertensives without hypertension medication [53].

While the pathophysiologic mechanisms illustrating the cardiotoxic effects of heavy drinking are well-described [10–12], those explaining the protective role of light drinking against the risk of HF are still obscure. Previous studies have demonstrated the beneficial effects of consuming alcohol in moderation on HDL levels [54] and insulin sensitivity [55]. Yet, adjusting for HDL and FBG in the current study did not affect the results. In addition, it was suggested that alcohol consumption might protect from incident HF by reducing CHD risk [14, 17, 19]. The US Kaiser Permanente study revealed a significant association between heavy drinking and increased risk of non-CHD-HF and apparent protection against CAD-HF risk [17]. Due to the limited number of participants with preceding stroke or CHD in the Suita Study, we could not stratify the results by this variable. Though, when we repeated the analysis after excluding those with preceding stroke or CHD, the association between heavy drinking and HF became stronger. Besides, a positive linear association between the amount of consumed alcohol and HF risk was noticed only among men with no preceding stroke or CHD; each 23 g increment in daily alcohol consumption was associated with a 19% increase in HF risk. However, the preceding stroke or CHD did not materially change the results and the preceding stroke or CHD variable did not interact with the association between alcohol consumption and HF risk in both sexes. Thus, we assume that cardiac ischemia did not solely explain the association between heavy drinking and HF risk. According to the sick quitter hypothesis, it could be speculated that some former drinkers who quit drinking for medical conditions might have inappropriately defined themselves as never-drinkers [32, 56]. Many studies reported an increased HF risk in former drinkers [17, 21, 22].

The association between heavy drinking and HF risk in the Suita Study was more pronounced among overweight/obese and ever-smoking men. Both obesity and smoking are major risk factors for CVD, including HF [57–59]. Our findings suggest a combined effect of alcohol consumption and other traditional CVD risk factors, such as obesity and smoking, on HF risk.

The strengths of the Suita Study are exploring the association between alcohol consumption and HF risk in a representative Asian population, applying a prospective design with frequent follow-ups, and controlling the results for most potential confounders. Still, some limitations should be addressed. First, although HF is typically diagnosed using a combination of clinical symptoms and echocardiography findings [41], we determined HF during follow-up using BNP levels or other physicians’ diagnoses. However, BNP has high diagnostic accuracy and BNP ≥ 100 pg/mL is likely to indicate HF [41]. Besides, previous population-based cross-sectional studies showed a strong association between heavy drinking and high BNP levels [60, 61]. Second, age, weight, and arrhythmia can affect BNP levels [62], suggesting misclassification. Though, adjusting for these variables did not materially change the results. Third, the absence of echocardiography findings did not allow us to investigate the relationship between alcohol consumption and HF subtypes. Fourth, alcohol consumption was self-reported; therefore, participants might have underreported the amount of their consumption. Though, since the baseline analysis showed a positive correlation between the amount of alcohol and HDL, a biological marker of alcohol consumption [63], this possibility is unlikely. Fifth, we did not stratify the results by the type of alcoholic beverage. However, the EPESE study showed similar associations with incident HF in beer, wine, and liquor [14]. Sixth, compared to the analysis group, those who lost to follow-up were significantly older and had higher proportions of hypertension, a cardiac murmur or valvular disease, arrhythmia, and preceding CVD, suggesting that they were more likely to develop HF.

Our meta-analysis had several strengths. First, we confined the meta-analysis to studies that assigned light or moderate drinkers as a reference group to avoid a potential spurious insignificant association between alcohol consumption and HF risk if never drinkers were assigned as a reference group. Second, all included studies used high cut-offs of alcohol consumption to define heavy drinking (at least three drinks/day). Further, the included studies were of good quality and had a prospective cohort design to avoid recall bias and imply a temporal association. Finally, our meta-analysis did not show publication bias, yet this possibility cannot be entirely excluded. It should be noted that the non-drinkers’ group in BRHS included former and never-drinkers; therefore, we used the term alcohol abstinence for the meta-analysis. Still, removing BRHS from the meta-analysis did not change the results.

The Suita Study and the meta-analysis reported a J-shaped association between alcohol consumption and HF risk. Alcohol consumption appears to have a complex relationship with incident HF. While heavy drinking may contribute to HF development, light drinking is not cardiotoxic and can even have a protective role against HF. Besides, the Suita Study showed that the impact of heavy drinking on HF was augmented among ever smokers and overweight/obese people, suggesting that these groups should be prioritized in screenings and other preventive approaches. Heavy drinking should be involved in clinical risk scores predicting HF.

## Abbreviations

ARIC: Atherosclerosis Risk in Communities Study
BMI: Body mass index
BNP: Brain natriuretic peptide
BRHS: British Regional Heart Study
CALIBER: ClinicAl research using LInked Bespoke studies and Electronic health Records
CHD: Coronary heart disease
CHS: Cardiovascular Health Study
CI: Confidence interval
CLEIA: Chemiluminescent Enzyme Immunoassay
COSM: Cohort of Swedish Men
CVD: Cardiovascular disease
EPESE: Established Populations for the Epidemiologic Study on the Elderly program
FBG: Fasting blood glucose
HDL: High-density lipoprotein
HF: Heart failure
HR: Hazard ratio
HUNT: Nord-Trøndelag Health Study
JACC: Japan Collaborative Cohort Study
JHFS: Japanese Heart Failure Society
Metafor: Meta-Analysis Package for R
NCVC: National Cerebral and Cardiovascular Center
NOS: Newcastle-Ottawa Quality Assessment Scale
PHS I: Physicians’ Health Study I
SHEEP: Stockholm Heart Epidemiology Program
SMC: Swedish Mammography Cohort

## Supplementary information

**Additional file 1:** **Supplementary file 1:** Differences between analysis and loss to follow-up groups. **Supplementary file 2:** Search strategy. **Supplementary file 3:** Risk of bias assessment using the Newcastle-Ottawa Quality Assessment Scale. **Supplementary file 4:** The impacts of removing studies one by one and combining the remaining studies. **Supplementary file 5:** Funnel plot showing publication bias in studies that assessed the association between heavy drinking and heart failure risk. **Supplementary file 6:** Funnel plot showing publication bias in studies that assessed the association between alcohol abstinence and heart failure risk.

## Declarations

### Availability of data and materials

Available upon reasonable request.
