# Supplementary material for: Alcohol consumption and the risk of heart failure: the Suita Study and meta-analysis of prospective cohort studies
Source: Environ Health Prev Med. 2023 May 3;28:26. doi: 10.1265/ehpm.22-00231 (PMC10188284; doi:10.1265/ehpm.22-00231)
Supplement: Supplementary file 1 — Additional file 1: Supplementary file 1: Differences between analysis and loss to follow-up groups. Supplementary file 2: Search strategy. Supplementary file 3: Risk of bias assessment using the Newcastle-Ottawa Quality Assessment Scale. Supplementary file 4: The impacts of removing studies one by one and combining the remaining studies. Supplementary file 5: Funnel plot showing publication bias in studies that assessed the association between heavy drinking and heart failure risk. Supplementary file 6: Funnel plot showing publication bias in studies that assessed the association between alcohol abstinence and heart failure risk. [file ehpm-28-026-s001.docx]

**Supplementary file 1: Differences between analysis and loss to follow-up groups**

| Characteristics | | Men | | Women | |
| --- | --- | --- | --- | --- | --- |
|  |  | Analysis | Loss to follow-up | Analysis | Loss to follow-up |
| Frequency | | 1,149 | 238 | 1,563 | 297 |
| Age, years* | | 67.1±10.2 | 70.6±11.5 | 65.5±10.0 | 70.8±11.9 |
| Body mass index, kg/m^2^* | | 23.4±2.8 | 22.9±3.3 | 22.3±3.3 | 22.4±3.9 |
| Smoking, % | Never | 25.9 | 21.9 | 89.5 | 85.2 |
|  | Former | 50.6 | 53.4 | 5.8 | 7.7 |
|  | Current | 23.5 | 24.8 | 4.7 | 7.1 |
| Alcohol consumption, % | Never | 29.6 | 35.3 | 74.4 | 76.4 |
|  | Light | 18.5 | 17.2 | 15.9 | 17.2 |
|  | Moderate | 25.3 | 24.4 | 7.0 | 4.0 |
|  | Heavy | 26.6 | 23.1 | 2.8 | 2.4 |
| Systolic blood pressure, mmHg* | | 130.0±18.1 | 132.6±21.4 | 125.1±20.3 | 130.1±21.5 |
| Diastolic blood pressure, mmHg* | | 80.3±10.3 | 80.2±11.5 | 74.9±11.1 | 76.0±11.0 |
| Hypertension medication, % | | 32.0 | 37.0 | 25.8 | 39.4 |
| Fasting blood glucose, mg/dL* | | 108.1±24.1 | 107.7±26.6 | 99.0±13.9 | 101.6±16.6 |
| Glomerular filtration rate, ml/min/1.73 m^2^* | | 74.3±15.6 | 71.3±17.8 | 77.2±15.7 | 73.4±17.0 |
| High-density lipoprotein-cholesterol, mg/dL* | | 55.9±14.8 | 55.5±14.6 | 65.8±14.9 | 63.8±15.7 |
| Lipid-lowering agent, % | | 14.4 | 16.8 | 21.8 | 24.9 |
| Cardiac murmur or valvular disease, % | | 6.4 | 8.0 | 7.6 | 14.5 |
| Arrhythmia including atrial fibrillation, % | | 15.5 | 19.3 | 12.4 | 14.8 |
| Preceding stroke or coronary heart disease, % | | 8.4 | 13.5 | 4.2 | 8.4 |

*Mean±standard deviation for all such variables

**Supplementary file 2: Search strategy**

**PubMed**

("ethanol"[MeSH Terms] OR "ethanol"[All Fields] OR "alcohol"[All Fields] OR "alcohols"[MeSH Terms] OR "alcohols"[All Fields]) AND ("heart failure"[MeSH Terms] OR ("heart"[All Fields] AND "failure"[All Fields]) OR "heart failure"[All Fields])

**Scopus**

TITLE-ABS-KEY ( alcohol AND heart AND failure )

**Supplementary file 3: Risk of bias assessment using the Newcastle-Ottawa Quality Assessment Scale**

| Item | BRHS | CALIBER | COSM | SMC | 83 cohorts | Suita |
| --- | --- | --- | --- | --- | --- | --- |
| Representativeness of the exposed cohort | * | * | * | * | * | * |
| Ascertainment of exposure | * | * | * | * | * | * |
| Selection of the non-exposed cohort | * | * | * | * | * | * |
| Demonstration that the outcome of interest was not present at the start of the study | * | * | * | * | * | * |
| Comparability | ** | ** | ** | ** | * | ** |
| Assessment of outcome |  | * | * | * | * |  |
| Follow-up was long enough for outcomes to occur | * | * | * | * | * | * |
| Adequacy of follow-up of cohorts | * | * | * | * | * | * |
| Overall (total number of asterisks) | 8 | 9 | 9 | 9 | 8 | 8 |

The possible overall scores range between 0 and 9

**Supplementary file 4: The impacts of removing studies one by one and combining the remaining studies**

| Heavy drinking and heart failure risk | | |
| --- | --- | --- |
| Removed study | HR (95% CI) | *I*^2^ |
| BRHS | 1.36 (1.13, 1.63) | 51.81% |
| CALIBER | 1.44 (1.21, 1.72) | 18.72% |
| COSM | 1.35 (1.09, 1.67) | 51.52% |
| SMC | 1.40 (1.19, 1.64) | 40.32% |
| 83 cohorts | 1.32 (1.06, 1.64) | 37.08% |
| Suita | 1.33 (1.13, 1.57) | 43.28% |
| Alcohol abstinence and heart failure risk | | |
| Removed study | HR (95% CI) | *I*^2^ |
| BRHS | 1.20 (1.03, 1.40) | 56.53% |
| CALIBER | 1.13 (9.94, 1.35) | 37.12% |
| COSM | 1.15 (0.95, 1.40) | 60.09% |
| SMC | 1.26 (1.08, 1.45) | 25.88% |
| Suita | 1.19 (1.03, 1.37) | 53.61% |

Test for Funnel Plot Asymmetry: Z = -0.392, p-value = 0.695

**Supplementary file 5: Funnel plot showing publication bias in studies that assessed the association between heavy drinking and heart failure risk**

Test for Funnel Plot Asymmetry: Z = -0.743, p-value = 0.457

**Supplementary file 6: Funnel plot showing publication bias in studies that assessed the association between alcohol abstinence and heart failure risk**
